# Dangerous practices in a hemodialysis unit in Vietnam identify from mixed methods

**DOI:** 10.1186/s12879-017-2290-3

**Published:** 2017-03-01

**Authors:** Minh Cuong Duong, Mary-Louise McLaws

**Affiliations:** School of Public Health and Community Medicine, UNSW Medicine, UNSW Australia, Level 3 Samuels Building, Sydney, NSW 2052 Australia

**Keywords:** Hemodialysis, Infection control non-compliance, Glove use, Hand hygiene, Barriers, Healthcare workers

## Abstract

**Background:**

Non-compliance with infection control practices poses a serious risk to patients receiving chronic hemodialysis. We aimed to identify the type and frequency of non-compliance with infection control practices in a hemodialysis unit in Vietnam where a large outbreak of hepatitis C infection had occurred.

**Methods:**

Mixed methods approach included observations and discussions of non-compliance with all 12 nurses at the Hemodialysis Unit, District-6 Hospital in Ho Chi Minh City. Observations of nursing care activities were made between September 2013 and January 2014. Compliance with hand hygiene and glove use during nursing care activities were classified according to the potential for a serious risk of transmission of infection and reported as percentages. Each nurse was expected to provide 11 nursing care activities to three patients assigned per hemodialysis sessions. Activities were to be given on an individual patient-centered care basis, that is, one patient was to receive all 11 activities by their assigned nurse. On completion of the observations all nurses were enrolled in a focus group where observed non-compliance was discussed and transcripts were examined for themes.

**Results:**

Hand hygiene compliance rate was low (27%, 95%CI 25%-28%, 1633/6140) regardless of classification of seriousness of risk from this breach. Although glove use (76%, 95%CI 74-78%, 1211/1586) and other personal protective equipment use (81%, 95%CI 78%-83%, 773/959) were high gloves were observed to be reused with multiple patients during a single nursing care activity provided to consecutive patients. Nurses explained the breakdown of providing nursing care activities on an individual patient-centered basis was a response to limited supply of gloves and hand hygiene facilities and was exacerbated by nursing being co-opted by overly demanding patients to provide services without delay.

**Conclusions:**

The adaption by the nurses to provide 11 single care activities to multiple consecutive patients in the absence of changing gloves and low hand hygiene compliance was potentially the central risk factor that facilitated the hepatitis C outbreak. Patient-centered care needs to be enforced to minimize multiple nurse-patient contacts that are associated with non-compliance classified as serious risk of infection transmission. Nurse empowerment to resist unreasonable patient demands may also be pivotal to assisting their compliance with hand hygiene and single patient-centered care. An audit program to measure infection control resources and practices may facilitate enforcement of the guidelines.

## Background

Viral hepatitis C (HCV) and B (HBV) infections remain a threat to patient safety for those patients on long-term hemodialysis treatment worldwide [1, 2]. Several high risk activities have been identified as being related to patients’ lifestyle and hemodialysis treatment [1, 3–6] and include serious breaches in infection control practice resulting in several viral hepatitis outbreaks in hemodialysis units worldwide [7–13]. Yet, such outbreaks should be precluded with the adherence to standard and contact precaution guidelines [14].

HCV and HBV infections are endemic in the general Vietnamese community [15, 16]. Also, data available since 1995 suggest the prevalence of HBV in patients in hemodialysis units across Vietnam ranges from 4.9 to 12.1% [17–19] while the prevalence of HCV ranges from 15.4% to a high level of 52.7% [17, 18, 20, 21]. Vietnamese national infection control and prevention recommendations were issued in 1997 [22] and have been regularly updated [23–25]. Yet, infection control practices have been reported to be suboptimal in general tertiary hospitals nationwide [26–28]. There is a trend to decentralize hemodialysis services out of tertiary hospitals to become provincial services without targeting training in infection control guidelines for these satellite facilities. We conducted a sero-prevalence survey in one of the largest satellite hemodialysis unit in Ho Chi Minh City and reported HCV (6%) and HBV (7%) were 7.5 times and 1.9 times respectively higher than the rates in the corresponding population in the same city [6]. This level of sero-prevalence suggested there were serious infection control and prevention challenges in these low-resource hemodialysis settings [6]. Indeed, a large outbreak of HCV infection associated with an unknown infection control breach was identified in this hemodialysis unit [29]. In September 2013 the unit had 119 patients of whom nine were HCV positive and nine were HBV positive. *Between the next testing cycle after September 2013 and up to and including January 2014, 128 patients received hemodialysis including nine who had a HCV infection previously identified and one who had HCV infection previously identified remained a patient in the unit while 12 patients became newly infected with HCV. Of the 12 new cases of HCV infections 11 were detected during a cluster outbreak between September and November 2013 and one case was detected between November 2013 and January 2014* [29]*. The two-floor unit had 15 hemodialysis machines (six on the ground floor and nine on the first floor) including dedicated machines for HCV and HBV infected patients. Non-concordant patients were identified to have shared these dedicated machines during September 2013 and January 2014 however machine sharing was ruled out as a likely causal factor for the outbreak* [29]. In the presenting study we report our observations of breaches in infection control and prevention at the same hemodialysis unit and report the seriousness and type of noncompliance to identify the common barriers to correct infection control practices for the prevention of HCV and HBV.

## Methods

The study was approved by the UNSW Australia Human Research Ethics Committee (reference HC12363), Ho Chi Minh City Health Service (reference 3242/SYT-VP) and District 6 Hospital (reference 223/TB-BV) authorities. All study participants provided written informed consent. The hemodialysis unit was located in a district level hospital in Ho Chi Minh City. A mixed methods approach comprised observations of all 12 nurses employed at the unit commenced between October 2012 and December 2014 for compliance with infection control practices without prior notice of the observations. In December 2014, after the completion of all observations focus group discussions (FGD) were held and all nurses were consented and provided their age, gender, months of nursing experience and infection control training. Data analysis of observations and FGD were delinked and the clinic director was unaware of which staff was observed and who attended the FGD.

### Setting of the study

#### Nurse-patient contact and patient condition

There were four treatments sessions daily: morning, noon, afternoon, and evening. In accordance with local policy the ratio of nurses assigned to patients is 1:3*.* The two-floor unit had 15 hemodialysis machines (6 on the ground floor and 9 on the first floor) including dedicated machines for HCV and HBV infected patients. In direct contradiction to the guidelines [30] medication preparation and clean supplies occurred at the nurses’ stations which were co-located with hemodialysis machines in the treatment room on each level. Stored at each nurses’ station were: a box of non-sterile gloves, alcohol based hand rub (ABHR) and pure alcohol dispenser that was supplied as a cheaper version of commercial ABHR. There were four handwashing basins in the unit. One handwashing basin was located at either end of each corridor approximately 9 m from the first bed. The remaining two basins on the ground floor were located in the doctor office and in the restroom outside the treatment room [29].

Nurses had 11 care activities that include six patient contact activities and five environmental contact activities (Table 1). The direct patient care activities comprised frequent and less frequent practices. Four frequent activities were: (1) weight measurements before and after hemodialysis (2) blood pressure measurement at 30 min intervals totaling approximating 6 measurements per patient per session (3) venipuncture and connection of the patient to the hemodialysis machine and (4) disconnection of the patient from the machine. The remaining two less frequent practices were: (5) wound dressing that included caring for patients with leg and feet ulcers due to diabetes related peripheral vascular disease [31] and (6) manipulating and managing patients’ bloodlines and ruptured dialyzer. The environmental contacts included (7) preparation of dialysis material such as heparin as patients must be anti-coagulated during hemodialysis using an injection of low dose heparin solution diluted in normal saline to obtain the recommended low concentration as local guideline; (8) preparation of the machine (9) administration of heparin (10) cleaning the dialysis room and (11) disposal of waste material.

**Table 1:** Classification of 11 patient-care activities

Classification	Activities
Common direct patient care activities	Weighing patients Blood pressure measurement Puncture and connection Disconnecting patient
Less common direct patient care activities	Wound dressing Manipulating patients’ bloodline
Environmental contacts	Preparation of dialysis material Preparation of hemodialysis machine Administrating heparin Cleaning room Disposal of material

Diabetes and hypertension co-morbidities were prevalent in our patient population [32] and blood pressure measurements could not be performed manually using mercury sphygmomanometer and a stethoscope because many of these patients had peripheral vascular disease. Therefore, nurses were required to use their fingers to estimate the radial pulse systolic blood pressure [33]*.*

### Study design

#### Observations of compliance

To establish a sample size of observations the expected frequency of breaches was set at 50% giving 385 observations required. Between September 2013 and January 2014, 11 care activities were observed that required hand hygiene, non-sterile glove use and personal protective equipment (PPE) in accordance with international best practice [14]. The audit tool used had been developed and previously tested elsewhere [34, 35] with modifications to meet the specific characteristics of the study unit and the current application of World Health Organization (WHO) ‘My 5 Moments for Hand Hygiene’ in a hemodialysis setting [36]. One researcher (MCD) kept field notes. Patients were routinely scheduled to receive treatment during one of three daytime hemodialysis sessions and an evening session was available to only those patients who could not receive treatment during day sessions. Nurses were randomly selected in each 30-min observation session to be observed in the morning (139), noon (100), afternoon (107) and evening (44) (Table 2). All observation sessions were performed during the absence of the Head of unit (HOU). Nurses 6 and 9 could not be observed during night sessions because neither worked at night.

**Table 2:** Number of observation by session and nurse

Nurse	Morning	Noon	Afternoon	Evening
Nurse 1	17	12	8	1
Nurse 2	11	7	11	3
Nurse 3	10	6	9	5
Nurse 4	15	10	6	3
Nurse 5	16	6	11	4
Nurse 6	15	10	9	0
Nurse 7	6	12	12	4
Nurse 8	11	4	8	5
Nurse 9	8	6	7	0
Nurse 10	12	8	9	6
Nurse 11	8	10	9	8
Nurse 12	10	9	8	5
Total	139	100	107	44

Breaches in the 11 previously listed care activities were presented as proportions, with 95% confidence interval (95%CI) for proportions, and classified for level of seriousness of breach (serious, moderately serious and less serious) based on a likelihood of blood exposure.

### Focus group discussions about breaches in infection control

Semi-structured interviews were developed for a one-hour focus group discussion in December 2014 in accordance with accepted methodology [37–39] and included knowledge about the implementation of infection control practices and then reflections on our observations of their infection control breaches. Interviews took place away from physicians to ensure confidentiality [39]. Discussion for each breach continued until discussion reached saturation. Prompting items and discussions were conducted in Vietnamese and audio tape recorded. The recordings were transcribed verbatim in Vietnamese and translated into English. Transcripts were analyzed manually using a thematic approach and analyzed for group interaction along with field notes [40, 41]. During the first reading of the transcripts the comment function in WinWord was used to code themes and write comments. By applying the constant comparison approach of the same code to all transcripts codes were categorized into themes [42] that were then reviewed (MLM) for consensus.

## Results

### Observations of compliance

Observations of 12 nurses were made during 390 treatment sessions. The mean age of the nurses was 26 years (Standard Deviation (SD) 4) and age ranged from 22–35 years. Most (75%, 9/12) nurses were female who had 31 months (SD 27, range 9–102 months) of nursing experience. All nurses reported to have received infection control training during orientation with an annual refresher course.

Care provided by nurses was activity-based rather than individual patient-centered. Each nurse was assigned to care for three patients. However, nurses were observed to provide the same nursing activity (outlined in Table 1) to all patients on their floor: 6 patients on the ground floor and 9 patients on the first floor. A single nurse would perform a clinical care activity, such as blood pressure measurement, to discordant patients during a treatment session (field notes). This resulted in each nurse having direct patient contacts with 12-18 patients to perform up to four frequent activities on each patient over two treatment sessions during an average 8-h shift. Bottles of pure alcohol for hand rub was observed in the rooms and the WHO formula for locally produced hand rub [43] were not available (field notes) and none of the nurses were aware of this formula.

Hand hygiene compliance was low at 27% (1633/6140, 95%CI 25-28%). Compliance with hand hygiene associated with potentially serious infection control procedures included before (6%) and after (91%) wound dressing, before (69%) and after (69%) the manipulation of a patient’s bloodline, before (29%) and after (37%) connection, before (24%) and after (37%) disconnection, before (8%) and after (16%) heparin injection, before preparation of dialysis material (55%) and after disposal of material (29%) (Table 3). Compliance with moderately serious infection control activities included before (26%) and after (28%) the preparation of the dialysis machine and before (8%) and after (17%) blood pressure measurement. Compliance with less serious activities included before (10%) and after (30%) weighing patients and cleaning the room (23%).

**Table 3:** Compliance with infection control practices associated with 11 patient-care activities

Activities	% [95%CI] (n/N)		
	Hand hygiene before	Hand hygiene after	Use of gloves
Serious breaches if not adherent to infection control practices			
Preparing dialysis material	55 [51–60] (253/456)		
Puncture and connection	29 [25–33] (157/543)	37 [33–41] (201/543)	60 [55–64] (324/543)
Administrating heparin	8 [6–12] (31/372)	16 [12–20] (58/372)	
Disconnecting patient	24 [20–29] (101/416)	37 [33–42] (156/416)	86 [83-89] (360/416)
Wound dressing	6 [2–20] (2/33)	91 [76-97] (30/33)	100 [90-100] (33/33)
Disposal of material		29 [25–34] (114/390)	75 [71-79] (294/390)
Manipulating patient blood line	69 [56-79] (40/58)	69 [56-79] (40/58)	93 [84-97] (54/58)
Moderately serious breaches if not adherent to infection control practices			
Preparation of the machine	26 [22–30] (107/412)	28 [24–33] (117/412)	
Blood pressure measurement	8 [6–10] (51/663)	17 [14–20] (111/663)	
Less serious breaches if not adherent to infection control practices			
Weighing patient	10 [5–19] (8/77)	30 [21–41] (23/77)	
Cleaning room		23 [17–30] (33/146)	100 [97-100] (46/146)

Glove use was high (76%, 95% CI 74-78%, 1211/1586). Glove use compliance was 100% for wound dressing and 100% during cleaning of the dialysis room, 93% for manipulation of patients’ blood lines, 86% for disconnection, 75% for disposal of material, and 60% for venipuncture and connection process (Table 3).

PPE use was high (81%, 95%CI 78-83%, 773/959) for all possible PPE opportunities and remained high for puncture and connection (82%, 95%CI 79-85%, 448/543) and disconnection (78%, 95%CI 74-82%, 325/416) (not shown in Table 3).

### Focus group discussions about breaches in infection control

All 12 nurses participated in focus group discussions. Three main themes were identified as barriers to habitual and appropriate hand hygiene and PPE use including glove.

1. Limited supplies

The supply of gloves was strictly controlled. *“The hospital requires us to not use gloves excessively…to reduce the hospital expenditure.”* An internal policy for the number of gloves to be used per patient was, *“Three pairs of gloves for each patient.” “Three pairs of gloves are the maximum number of gloves for a whole hemodialysis session of one patient from arriving at the clinic until finishing his treatment.”* Because of the glove quota nurses learnt to preserve gloves for activities that they perceived to pose the highest risk of blood exposure “*We tend to only use gloves for priority purposes, such as wound dressing, puncture and connection, and disconnection*” Nurses thought the glove restriction policy was *“strict and rigid”* and *“[it] doesn’t ensure patient safety.”*

The division of care for the same procedure to multiple patients consecutively in the same treatment session meant a single pair of gloves was re-used on different patients for multiple contacts. Gloves were also not provided in a variety of sizes. *“Gloves are supplied [in batches] in one size at a time [so] the size will change each [batch].”* The limited availability of sizes discouraged habitual glove use as indicated by the guidelines. “*The size of gloves doesn’t fit us…our hands are too small to fit the [supplied] gloves.”* Nurses also complained that the supplied gloves torn easily *“These gloves are torn easily so that the number of available gloves doesn’t meet our need.”*

The poor quality of the arteriovenous fistula (FAV) in some patients [44, 45] was a common barrier to nurses’ glove use during FAV manipulation with nurses palpating veins prior to inserting needles with ungloved hands (field notes). Nurses did not don gloves to perform palpation and needle use even when they understood gloves were indicated “G*loves are definitely needed when contacting with blood*.” But when asked why they inserted needles into FAV without wearing glove nurse said they were reluctant to learn to accommodate performing this procedure with gloves “*There are some patients whose FAVs are very difficult to identify. We have to use our bare hands to feel the veins. We can’t do it with gloves, especially with thick gloves*.”

Nurses have not attempted to master glove use for other similar activities and understood that needle stick injury to ungloved hands will increase their risk of blood-borne virus exposure. “*There are some patient-care activities, such as measuring blood pressure, and checking pulse rate and its characteristics that can be performed better if we use bare hands*.” and *“I do wear gloves when injecting heparin into the bloodline. We just don’t use gloves when diluting heparin. Injecting heparin (into the bloodline) means we contact with blood and thus we have to wear gloves.”*

During the provision of a single nursing care activity to multiple patients “*we re-use the gloves with the next patient*”. They were unaware of the risk of cross-transmission to the consecutive patients from reusing their gloves. “*The common practice is that we won’t wash our hands if we use gloves. And if we don’t use gloves, we will wash our hands.*” Nurses have developed their own interpretation of indications for hand hygiene: “*We don’t handwash (after the same patient care activity) if we think (the patients) are clean when we don’t touch patients’ blood and secretion*.” When they did change gloves after disposing of dialysis material hand hygiene was not performed. “*Actually we rarely handwash when we dispose dialysis material. We just need to change gloves*.” Nurses did wear gloves during caring wounds and when asked what indicated hand hygiene they believed “*It depends on the severity of the wounds*”.

Gloves were often used during the preparation of dialysis machine to replace hand hygiene. “*In practice, if the machine is visually clean, we just need to change our gloves (after finishing touching that machine).*” When asked to define a ‘clean machine’ they talked about the lack of visible contamination “*It means the machine isn’t visually contaminated with blood*.” *The quality of hand hygiene* products discouraged hand hygiene becoming habitualized while *cost of the products* discouraged *supply*. “*There’s a limited quota for the supply of ABHR solution [and] not enough for us to wash hands frequently.*” *We usually use pure alcohol for rapid hand hygiene. It’s not [a commercial brand] which is the most common ABHR in hemodialysis units.*” The rationale for the replacement of ABHR with pure alcohol was in the absence of their knowledge about the WHO recommended local production [43]. The pure alcohol had a severe drying effect on their hands that impeded compliance with further hand hygiene. Scarce handwashing facilities impacted on soap and water washing. “*We don’t have enough sinks in the unit and the current sinks are located far away from the dialysis room*.” “*Sometimes the sinks don’t work properly. We have to go upstairs or downstairs (to wash our hands). This is extremely time-consuming.*” Nurses suggested the appropriate sink location would be in the treatment room. “*We’ll wash our hands more frequently [if] the sink is just next to us.”* Lack of hand towels also discouraged handwashing because wet hands made wearing gloves, either appropriately used or not, difficult. “*Hand towels aren’t available*” “*The gloves easily tear if we wear after washing our hands*” “*The glove powder will stick to our hands when wearing gloves”* As a result nurses routinely donned gloves without prior hand hygiene “*We wash our hands while our hands are still covered by gloves*.” Nurses believed adapting practices reduced the drying effect of their hands and accommodated the limited glove supply.

Supplies of masks were chronically low “*There is one mask per day*” but there are a few exceptions when nurses replace their surgical mask such as “*reprocessing the dialyzers*”, “[if the] *mask has been dropped on the floor*”. The inadequate supply of masks resulted in an inappropriate re-use of masks when masks loose the protective properties when wet or handled multiple times with contaminated hands by the user [26].

(2) The patient factor

Of the patients attending the unit 17% had been previously identified as non-cooperative and verbally or physically abusive and 12% shortened their treatment sessions because they arrived late or discharged themselves early before completing a full hemodialysis session [46]. Local policy required patients to arrive prior to the commencement of their treatment session and patients who arrive late would demand immediate treatment, often becoming aggressive when they perceived their wait-time for nurses to perform hand hygiene with soap and water was excessive “*Patients will become uncomfortable and even angry if they have to wait [for us] for a long time*”. When access to catheters was required nurses felt they were often unable to perform hand hygiene even rapidly with ABHR. “*Sometimes when a patient [lying on the dialysis bed waiting for connection] becomes very tired [due to volume overload] he asks us to come to connect him [to dialysis machine] quickly…[but] I’ve just finished caring for another patient and I have to run to this tired patient as quickly as I can to help him.”* “*In those [critical] situations we don’t have time to handwash even with ABHR but we do wear gloves.*” “[because] w*e need to care for patients first*.” During these activities nurses did not perform hand hygiene between patients and instead wore gloves but did not change gloves between patients. Hand hygiene depended on the nurse’s perception of the patients’ personal hygiene rather than routine application of the guidelines for the prevention of cross-transmission between patients: *“We definitely need gloves if we touch patient [name removed]. That patient has poor personal hygiene”.*

The process of nurses providing care for a series of patients resulted in nurses using the same gloves on multiple patients and only performing hand hygiene once they had completed the same tasks on multiple patients. *“We tend to wash our hands once we complete the puncture and connection for all patients.”* “*We just want to finish the task fast” “habit plays an important role…. [and] time is sometimes just a reason for us not to wash our hands.”*

Like glove use, nurses’ use of masks and gowns was motivated by perceptions that a patient posed a risk to the healthcare workers: “[we don a clean mask before] *care for patients with poor personal hygiene.*”

(3) The boss

When the HOU was present the HOU would prompt the nurses “*She will remind us when she’s available at the clinic.*” “[but] s*he isn’t here at all the time*.” In the absence of the HOU none of the full-time and part-time physicians and nurse unit managers took responsibility for infection control compliance “*They [physicians and head nurse] focus on the patients’ conditions. They don’t even care whether nurses wash their hands or not.*” The absence of a national infection control audit program means the manager leads compliance but only when they are present.

## Discussion

It is accepted in high-resource settings that patient-centered care is the cornerstone of quality patient care and safety [47]. Yet, the provision of a single care activity provided to multiple patients, regardless of infection status, in our unit is likely to be the central risk factor that predisposed the outbreak of hepatitis [29]. Our observations have unveiled a culture of noncompliance with multiple basic infection control guidelines. These breaches occurred while providing a single care activity that should have been given to a single patient but was given to multiple consecutive patients. This practice of multiple patients receiving single care activity has possibly resulted from staffing challenges in a low-resource hemodialysis unit in Vietnam. We believe that it is likely that this practice of single care activity given to multiple consecutive patients was the most likely scenario for the transmission of HCV. Nurses were observed to perform hand hygiene and change gloves only after providing a single care activity to multiple patients in the belief that this practice will allow them to complete their tasks faster. Yet, this practice is highly likely to increase transmission to more patients than would not have occurred if nursing care had been single patient-centered and where each nurse was assigned a strict number of patients during any one shift. The effect of non-compliance across multiple patients facilitates the transmission of healthcare associated infection, such as hepatitis, when a serious breach occurs between discordant patients [48]. The suboptimal compliance rate with hand hygiene (37%) and proper glove use (33%) was reported to be associated with HCV cross-transmission among patients in a hemodialysis setting through environmental contamination [49]. Hand hygiene is considered to be the most effective tool to prevent these infections [50] yet the hand hygiene rate was observed to be unacceptably low for all potentially serious infection control procedures that pose a high risk of blood contamination. The transmission of bacteria via contaminated gloved hands has been found to be significantly more effective than contaminated hands [51, 52]. Non-compliance with hand hygiene and reuse of gloves with multiple patients may have facilitated the largest outbreak of HCV infection in our unit [29]. Nurses gave the limited supply of PPE as the main reason for the reuse of gloves and it is therefore imperative to financially support healthcare facilities to provide adequate quantities of PPE.

Several barriers to correct infection control practices that concur with other findings in developing countries included suboptimal provision of PPE materials and hand hygiene utilities [50, 53, 54]. In Vietnamese hospitals, inadequate supplies of masks resulted in re-use over one or more days and poor supplies of hand hygiene solutions was linked to poor hand hygiene [26, 55]. The cost-effectiveness of hand hygiene program in reducing healthcare associated infections in intensive care units in Vietnam have been well demonstrated [56]. A cost-effective ABHR was not supplied to our participants as they were not aware of the WHO ABHR formulation even though Vietnam is a signatory of the ‘My 5 Moments for hand hygiene’ global program [57]. This leads to the use of pure alcohol as an alternative to ABHR and therefore the development of a coping mechanism to prevent the drying effect of pure alcohol rub and limited supplies of PPE is not an effective prevention strategy [51].

Patients’ non-cooperation to treatment schedules and demanding behavior are obvious challenges to improving infection control compliance in a busy dialysis unit [58]. Nurses were afraid of making patients become even more aggressive if they were asked to wait while the nurse performed hand hygiene. Glove reuse and the provision of a single activity to multiple patients became a coping strategy rather than considering hand hygiene as a duty of care to all patients [55].

The effect of peer review improves basic infection control compliance such as hand hygiene [59] and our observations concur in that the absence of review auditing has enabled low compliance rate to become the norm. Decentralized satellite units need standardized infection control education programs and audit using a two-phase approach starting with the introduction of an infection control liaison nurse to every unit who will educate and provide confidential feedback [60]. But some current infection control guidelines developed by high-resourced countries may not be applicable to less-developed settings. WHO has developed infection control prevention for low-resourced hemodialysis settings [53] and the lack of awareness of the WHO ABHR formula implies the application of these guidelines is still a challenge.

The use of mixed-methods strengthened our understanding of the practices and barriers to infection control regulations that could not be wholly quantitatively observed. The bilingual researcher (MCD) checked transcripts for translation. This researcher was embedded in the unit from October 2012 to December 2014 to develop trust between staff and researcher [61, 62] while conducting patietn surveys prior to conducting focus group discussions. This trust may have reduced the Hawthorne effect often resulting from direct observations [63, 64]. A limitation of our study is member checking of themes with the nurses post analysis was not performed but observations of breaches were discussed with the participants during the focus group discussions.

We identified several correctable infection control lapses that we believe facilitated the outbreak of HCV infection. To prevent healthcare-associated HCV and HBC infections recommendations from our findings have been implemented at the study unit. The initial affordable action taken was that each nurse is now strictly required to care for their three patients assigned to them during a working shift. Proposed action plans have been also developed to improve nurse infection control education and increase the supply of PPE and nurse empowerment against unreasonable patient demands. The likelihood that our findings could be generalizable to other hemodialysis units is high as many units will be located in similar settings as the study unit in Vietnam and other comparable low-resourced countries.

## Conclusions

The outbreak of HCV infection at our hemodialysis unit is multifactorial. This study unveiled possibly the most important infection control breach associated with a large outbreak of HCV infection in the hemodialysis setting: the practice of applying a single nursing care procedure to multiple consecutive patients while breaching multiple infection control guidelines that included sub-optimal hand hygiene and reusing PPE. Nurse empowerment against unreasonable patient demands may be pivotal to improving compliance with simple infection control. An audit program to measure infection control resources and practices could facilitate enforcement of the guidelines and provision of gloves. To reduce the risk of transmission single patient-centered care and adherence to the 1:3 nurse to patient ratio must be an enacted and be enforced.

## Abbreviations

ABHR: Alcohol based hand rub
CI: Confidence interval
FAV: Arteriovenous fistula
FGD: Focus group discussion
HBV: Hepatitis B virus
HCV: Hepatitis C virus
HOU: Head of unit
PPE: Personal protective equipment
SD: Standard deviation
UNSW: University of New South Wales
WHO: World health Organization
